# Widely targeted metabolomics reveals the antioxidant and anticancer activities of different colors of *Dianthus caryophyllus*

**DOI:** 10.3389/fnut.2023.1166375

**Published:** 2023-05-19

**Authors:** Xuhong Zhou, Miaomaio Wang, Hong Li, Shilong Ye, Wenru Tang

**Affiliations:** ^1^Office of Science and Technology, Yunnan University of Chinese Medicine, Kunming, China; ^2^Open and Shared Public Science and Technology Service Platform, Traditional Chinese Medicine Science and Technology Resources in Yunnan, Kunming, China; ^3^Laboratory of Molecular Genetics of Aging and Tumor, Medical Faculty, Kunming University of Science and Technology, Kunming, China

**Keywords:** carnation flowers, edible flower, flower color, phytochemical composition, biological activity

## Abstract

Carnation is edible flower that has potent antioxidant properties and is used in traditional Chinese medicinal system and food industry. The phytochemicals responsible for these various proprieties, however, are not fully understood. Thus, in order to recognize metabolite diversity and variability in carnation flowers of different colors and to discover key metabolites that contribute to the differences in antioxidant and anticancer activities, widely targeted LC-MS/MS-based metabolomics analysis was conducted on purple, green, yellow, and white carnation flowers. We identified and chemically categorized 932 metabolites. Metabolic compounds varied significantly with flower color. Several flavonoids, organic acids, phenolic acids, and nucleotides and their derivatives were found to be specific differential metabolites in purple flowers. A total of 128 key differential metabolites were screened. The purple flowers were found to have the highest antioxidant and anticancer activities compared to the other colored flowers. Correlation analysis revealed that the 6-hydroxykaempferol-3,6-*O*-diglucoside, 6-hydroxykaempferol-7-*O*-glucoside, quercetin-3-*O*-sophoroside, and 2′-deoxyguanosine were found to be the major constituents of the antioxidant and anticancer activities. 2′-Deoxyguanosine has effective antiproliferative activity against A549 and U2OS cells for the first report. At the same time, the combination of 2′-deoxyguanosine with 6-hydroxykaempferol-3, 6-*O*-diglucoside, or quercetin-3-*O*-sophoroside have also been found to increase the antitumor activity of 2′-deoxyguanosine. These discoveries enrich information on the phytochemical composition of carnation of different colors and provide resources for the overall use and improvement of carnation flowers quality.

## 1. Introduction

The flower has always been an integral part of our culture and is considered to be a symbol of natural beauty in literature. Flowers are also cultivated for a variety of nutritional and biological properties (1–4). It can be used both for adding sensory properties (color, taste, aroma, etc.), for decorating dishes, and more recently for the functional properties of the antioxidant, anti-inflammatory and anticancer activity derived from its plant constituents (5–7).

It was found that flowers are an important source of antioxidants, and peonidin-3-O-arabinoside and cyanidin-3-O-arabinoside are the main components affecting the antioxidant activity of *Paeonia delavayi* flowers (8). Ferulic acid, (+)-catechin, isoquercitrin, and quercitrin have been shown to be potential bioactive compounds of 30 edible flowers such as *Lonicera japonica* Thunb., *Osmanthus fragrans*, *Rosa rugosa* Thunb, etc. for use as natural antioxidants (9). Loizzo et al. (10) found *Sambucus nigra* exhibited the highest radical scavenging activity in eight edible flowers, followed by *Hedysarum coronarium*. Flowers have been shown to have anticancer properties that prevent cancer cell proliferation, tumor invasion and angiogenesis (7). Recent studies have shown anticancer activity of *Tagetes erecta* petal extract against cancer cell lines using MTT (3-(4,5-dimethylthiazol-2-yl)-2,5-diphenyltetrazolium bromide) assay (11). Saffron was found to have significant antiproliferative and antitumorigenic properties in osteosarcoma cancer cells (12). Hydro-alcoholic extract of *Achillea wilhelmsii* C. Koch possessed growth inhibitory activity by altering the expression of genes associated with cell death and fought against cancer (13). The total flavonoid extracted from *Nepeta cataria* L. can interfere with miR-126 expression and regulate the PI3K-AKT signaling pathway in order to cope with the anticancer effects (14).

*Dianthus caryophyllus*, a member of the Caryophyllaceae family, is a species of plant native to Iraq and the Mediterranean region. Dried flower buds were used in the treatment of throat and gum infections, wound healing, and gastrointestinal illnesses (8). The scavenging effect of the volatile oil from the carnation flowers possessed scavenging effect. For example, the application of 400 ppm putrescine improved the quality of the carnation oil by increasing the content of eugenol, which has been shown to have potent antioxidant activity (15). Kaempferide triglycoside, a glycosylated flavonol from a carnation cultivar, has been shown to inhibit proliferation of native and estrogen receptor β overexpressing colon cancer cells through a mechanism that is not mediated by ligand-binding-dependent activation of the estrogen receptor. Kaempferide triglycoside affected HCT8 cell cycle progression by increasing the G0/G1 cell fraction and in estrogen receptor β overexpressing cells increased two antioxidant enzymes (16).

With the development of metabolomics, liquid chromatography and mass spectrometry have been applied to analyze the metabolite profiles of flowers and to detect variations in their composition (17–19). At present, studies on different flower colors of *D. caryophyllus* mainly focus on phenotype and gene studies, and there is little research on their functional activities. The aim of this paper was to investigate the chemical composition of carnation using a widely targeted metabolomics method and to identify the differentially accumulated metabolites (DAMs) of four different colored carnation flowers. Our results reveal the antioxidant, anticancer activities of four different colors of carnation flowers, in order to stimulate their consumption and develop innovative ingredients for future flower foods.

## 2. Materials and methods

### 2.1. Plant materials

**FIGURE 1:**
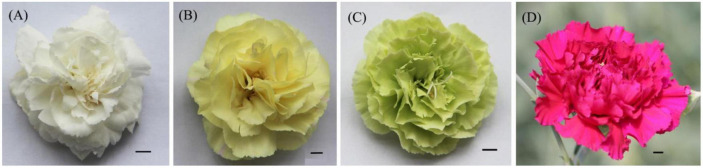
Phenotype of each colored flower. **(A)** White flower; **(B)**, yellow flower; **(C)** green flower; and **(D)** purple flower.

Four kinds of carnation materials with different colors, white flower (WF), yellow flower (YF), green flower (GF), and purple flower (PF), were used in this study (Figure 1). The plants were grown in Herbal Garden, Chenggong Campus, Yunnan University of Chinese Medicine. Three biological replicates were analyzed independently for each specific color. Petals were collected at random from six individuals and pooled for each biological replicate. All samples were collected on the same day, immediately placed in liquid nitrogen, and stored at −70°C until vacuum freeze-drying.

### 2.2. Ultra performance liquid chromatography and mass spectrometry

Using a vacuum freeze-dryer, biological samples were freeze-dried (Scientz-100F). The freeze-dried sample was crushed for 1.5 min at 30 Hz in a mixer mill (MM 400, Retsch). A total of 100 mg of lyophilized powder were dissolved in 1.2 ml of 70% methanol solution, vortexed six times for 30 s every 30 min and stored at 4°C overnight. Prior to UPLC-ESI-MS/MS analysis, extracts were centrifuged at 12,000 rpm for 10 min and then filtrated.

UPLC-ESI-MS/MS system was used to analyze sample extracts (UPLC, SHIMADZU Nexera X2; MS, Applied Biosystems 4500 Q TRAP). A C_18_ column (Agilent SB-C_18_, 2.1 mm × 100 mm, 1.8 μm) was used for the chromatographic analysis. By using the following elution gradient: 0–9 min, 5–95% B; 9–10 min, 95% B; 10–11.1 min, 95–5% B; 11.1–14 min, 5% B, the mobile phase was eluted with pure water containing 0.1% formic acid (A) and acetonitrile containing 0.1% formic acid (B). The column temperature was 40°C, the flow rate was 0.35 ml/min, and the injection volume was 4 μl.

The following were the ESI source operation parameters: ion source: turbo spray; source temperature: 550°C; ion spray voltage: 5,500 V (positive ion mode)/−4,500 V (negative ion mode); ion source gas I, gas II, curtain gas were set at 50, 60, and 25.0 psi, respectively; the collision-activated dissociation was high. Instrument tuning and mass calibration were carried out with solutions of 10 and 100 μmol/L polypropylene glycol in the QQQ and LIT modes, respectively. QQQ scans were obtained by MRM (Multiple Reaction Monitoring) experiments in which collision gas was set to medium. According to the metabolites eluted during each period, a particular set of MRM transitions were monitored.

### 2.3. Identification of metabolites and statistical analysis

Quantitative metabolite analysis was performed on the basis of the MVDB V3.0 Database of Wuhan Metware Biotechnology Co., Ltd. (Wuhan, China). Unsupervised principal component analysis (PCA) was carried out by the statistics function prcomp in R.^1^ The data were unit variance scaled prior to unsupervised PCA. The hierarchical cluster analysis (HCA) results for the samples and metabolites were presented in the form of heatmaps with dendrograms. Significant metabolite regulation between groups was determined by VIP ≥1 and log2FC absolute value (fold change) ≥1. VIP values were extracted from the output of OPLS-DA using the R package MetaboAnalystR, which contained score plots and permutation plots. Data were log transforms (log2) and mean centering prior to OPLS-DA. We conducted a permutation test (200 permutations) to avoid overfitting. The metabolites identified were annotated using KEGG compound database.^2^

### 2.4. Antioxidant activities

The 1,1-diphenyl-1-picrylhydrazino radical (DPPH) assay was carried out according to the method reported by Zhang et al. (20) with slight modifications. DPPH solution was used as substrate (0.15 mM) and divided into three groups: A: negative control group (160 μl methanol and 40 μl DPPH solution); B: experimental group (160 μl sample solution or VC and 40 μl DPPH solution); and C: experimental control group (160 μl sample and 40 μl methanol) were added sequentially to the 96-well plate. After 30 min of completely preparing the reaction was in the dark, the absorbance value were measured at 517 nm.

The free radical scavenging rate of DPPH was calculated using the following formula: (DPPH scavenging rate (%) = [A − (B − C)] / A × 100%).

Where A refers to negative control absorbance, B refers to experimental group absorbance, and C refers to experimental control group absorbance.

In this study, we referred to the method of 2,2′-azino-bis-3-ethylbenzthiazoline-6-sulfonic acid (ABTS) assay reported by Huang et al. (21). An equal volume of 7 mM ABTS solution was mixed with 2.45 mM potassium persulfate solution and stored in the dark at 4°C for 10–12 h, then diluted with methanol until the absorbance measured by a microplate reader at 734 nm reached 0.7 ± 0.05. Different concentration gradients were used to dilute the extracts. Blank group (100 μl methanol and 100 μl ABTS solution), experimental group (100 μl sample or VC and 100 μl ABTS solution), and experimental control group (100 μl sample and 100 μl methanol) were added sequentially to 96-well plates. After complete dark preparation of the reaction for 30 min, the absorbance value was measured at 734 nm. Three parallel experiments were set. The radical scavenging effect of ABTS was calculated according to the same equation described in the DPPH assay.

### 2.5. CCK-8 assay

For the detection of CCK-8, we referenced to the method reported by Zhao et al. (22). A549 cells and osteosarcoma cells (U2OS) were routinely grown in RPMI-1640 medium supplemented with 10% fetal bovine serum and 1% penicillin-streptomycin, digested and passaged, and plated in a 96 well plate supplemented with 1 × 10^4^ cells/well. The cells were cultured in a 5% carbon dioxide incubator for 24 h. After 24 h of drug treatment, 10 μl of the CCK-8 solution was added to each well, and culture was continued at 37°C for 1 and 2 h, respectively.

The absorbance was monitored at 450 nm. Cell viability was calculated using the formula: cell viability (%) = [(As − Ab) / (Ac − Ab)] × 100%.

Where As refers to the absorbance of the experimental well, Ac refers to the absorbance of the control well, and Ab refers to the absorbance of the blank well.

## 3. Results

### 3.1. Antioxidant and anticancer abilities of the different colored carnation flowers

**FIGURE 2:**
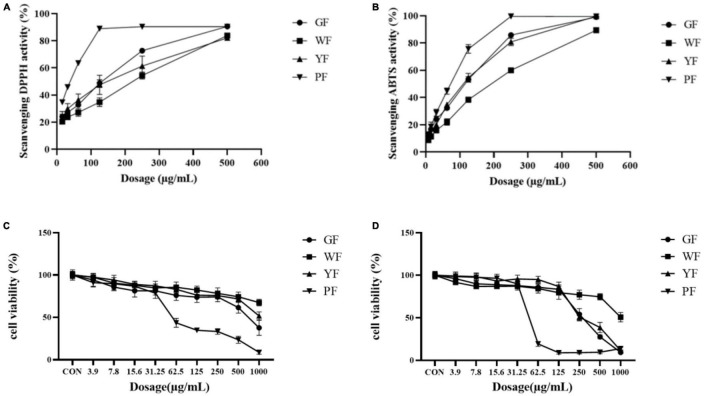
Scavenging DPPH activities **(A)** and ABTS activities **(B)** of total crude extract of green flower (GF), white flower (WF), yellow flower (YF), and purple flower (PF) measured using the DPPH assay. Each test was done in triplicate. Cell viability of methanol extract of GF, WF, YF, and PF against A549 **(C)** and U2OS **(D)** cancer cell lines.

2,2′-Azino-bis-3-ethylbenzthiazoline-6-sulfonic acid and DPPH assays were used to reveal the antioxidant activities of the different colored carnation flowers. As the data presented in Figure 2, the inhibitory effect of carnation flowers extracts on DPPH radicals was dose dependent. DPPH assays revealed that the antioxidant activities of the carnation flowers increased with the deepness of flower color. Overall, purple carnation flowers (IC_50_, 37.42 ± 0.20 μg/ml) were found to have higher antioxidant activities compared to carnation flowers of other colors. The antioxidant activities of green carnation flowers (IC_50_, 165.74 ± 2.17 μg/ml) was higher than that of white carnation flowers (IC_50_, 261.36 ± 9.31 μg/ml) and yellow carnation flowers (IC_50_, 202.52 ± 22.78 μg/ml). Antioxidant efficacy was lowest in white carnation flowers (Figure 2A). The DPPH free radical scavenging effect of carnation flowers were far from that of positive control Vc (IC_50_, 0.9723 ± 0.0346 μg/ml).

Antioxidant activities (IC50, μg/ml) were observed in purple flowers extract (50.43 ± 2.60 μg/ml), green flowers extract (77.12 ± 2.67 μg/ml), yellow flowers extract (83.12 ± 3.04 μg/ml), and white flowers extract (147.43 ± 2.15 μg/ml) using ABTS assays (Figure 2B). As shown in Figure 2B, all color flower extracts exhibited dose-dependent inhibition on the ABTS radical. The purple carnation flowers had the highest antioxidant efficiency, followed by the green flowers and yellow flowers, while the white carnation flowers had the lowest antioxidant efficiency. Antioxidant activities of carnation flowers using ABTS assays were lower that of positive control drug Vc (IC_50_, 2.813 ± 0.0627 μg/ml).

The methanol extract (3.9–1,000 μg/ml) was shown to have growth inhibitory activity against two utilized the human cancer cell lines (Figures 1C, D). The methanol extract of purple carnation flowers was most effective against both A549 and U2OS cells exhibiting both growth inhibitory. The IC_50_ potency order appears to be PF < GF < YF < WF (Table 1).

**TABLE 1:** IC_50_ values of the extracts of carnation of different colors.

No.	Sample	A549⋅IC_50_ (μg/ml)	U2OS⋅IC_50_ (μg/ml)
1	GF	≥500	271.2 ± 27.51
2	WF	≥500	≥500
3	YF	≥500	314.5 ± 38.34
4	PF	81.86 ± 7.289	47.53 ± 2.065

**FIGURE 3:**
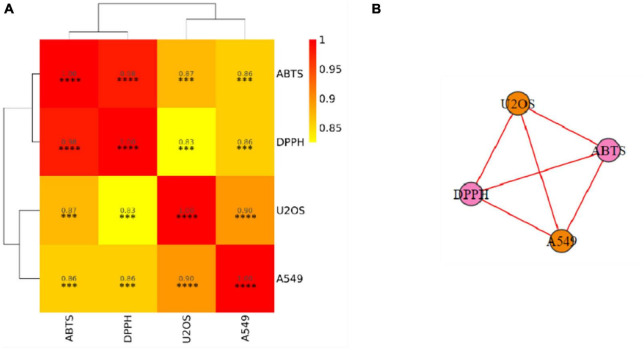
Associations among different indicators. **(A)** Correlation heatmap. **(B)** Correlation network. DPPH, DPPH scavenging activity; ABTS, ABTS scavenging ability; A549, A549 cancer cell lines inhibiting ability; U2OS, U2OS cancer cell lines inhibiting ability. The color represents the correlation value. The closer the value is to 1, the stronger the correlation between two duplicate samples is. Significant differences are indicated by an asterisk, ^***^P < 0.001, ^****^P < 0.0001.

In order to better elucidate similarities and differences between antioxidant and anticancer activities, a further correlation analysis was carried out and presented in Figure 3 in the form of the correlation heatmap and the correlation network. High and significant correlation coefficients indicated a high correlation between anticancer capabilities and different antioxidant capabilities.

### 3.2. Metabolite profiles of the different colored carnation flowers

**FIGURE 4:**
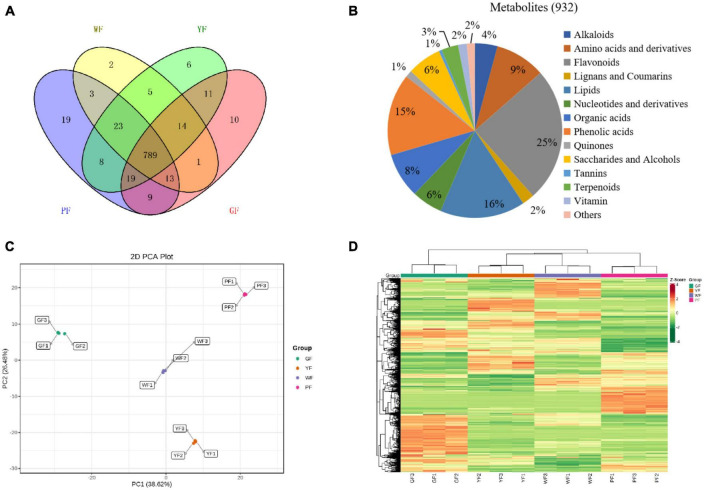
The diversity of 932 diverse metabolites in the purple, green, yellow, and white carnation flowers. **(A)** Distribution of the 932 metabolites in flowers of different colors; **(B)** classifying the 932 identified metabolites in carnation flowers; **(C)** group differences in metabolite profiles were revealed using principal component analysis (PCA); and **(D)** metabolites variation among groups were revealed using hierarchical cluster analysis (HCA). A column represents each sample, and each metabolite is shown in a row. Red represents higher metabolite abundances, while blue represents a lower metabolite abundances.

The metabolome analysis of white, yellow, green, and purple carnation flowers (WF, YF, GF, and PF) was performed to detect metabolites variability in carnation flowers of different colors using widely targeted metabolomics analysis method (Figure 1). We were able to structurally detect or annotate a total of 932 metabolites in carnation flowers (Supplementary Table 1). Of them, a total of 789 were common to WF, YF, GF, and PF (Figure 4A). These metabolites were classified into fourteen (8) categories, which were dominated by flavonoids (25%), lipids (16%), phenolic acids (15%), amino acids and derivatives (9%), organic acids (8%), saccharides and alcohols (6%), and nucleotides and derivatives (6%) (Figure 4B). The number of metabolites that were specific to WF, YF, GF, and PF were 2, 6, 10, and 19, respectively (Figure 4A). The PF specific metabolites included 17 flavonoids, 1 phenolic acid, and 1 organic acid (Supplementary Table 2).

Principal component analyses of samples aids in understanding general metabolic differences between groups as well as the degree of variability between samples within the same group. The overall PCA of each group and QC (quality control) samples showed different metabolite profiles in the WF, YF, GF, and PF carnation flowers (Supplementary Figure 1). The repeatability and reliability of this experiment were confirmed by the fact that three duplicate QC samples were closely grouped in the middle of the PCA plot. Figure 4C showed that the variability of metabolites in the different colored carnation flowers is also indicated by the PCA result. In addition, four groups of flower samples divided into different regions based on the first principal component (PC1) and the second principal component (PC2). PC1 (38.62%) and PC2 (26.48%) together accounted for 65.1% of the total variance. Results showed that PC1 isolated pigmented (YF and PF) and non-pigmented flower (GF and WF), suggesting that genetic variation strongly influenced the distribution of metabolites in both pigmented and non-pigmented carnation varieties. YF and PF, as well as GF and WF were predominantly isolated by the PC2, indicating significant differences in their metabolite profiles. As can be seen, 12 samples from the four materials were divided into four distinct clusters, indicating that each cluster had a distinct metabolite profile.

To detect patterns of metabolites in different clusters, we performed HCA on the heatmap. This resulted in four main clusters based on the differences in relative metabolite content, respectively (Figure 4D). These results suggests that the four groups have significantly different metabolic profiles. Each group was subjected to three biological replicates, indicating sufficient repeatability and high reliability of data between replications. Based on the PCA and HCA results, the four cultivars were divided into four distinct clusters, indicating significant differences in metabolite distribution between the clusters.

### 3.3. Identification of differential metabolites

We performed an OPLS-DA analysis to gain insight into the metabolic differences and identify differential metabolites between the groups. The score plots of the pairwise comparisons can be found in Supplementary Figure 2. High predictability (Q2) and strong goodness of fit (R2X and R2Y) were observed, suggesting that the metabolite profiles of the green, yellow, white, and purple carnation flowers were too different from one another. For example, the Q2 values between GF and WF, GF and YF, GF and PF, PF and WF, PF and YF, and WF and YF were found to be 0.993, 0.994, 0.997, 0.994, 0.993, and 0.991, respectively (Supplementary Figure 3). Metabolites with significant differences between each pairwise comparison were screened using the criteria of *P*-value < 0.05 and VIP ≥1. The results visualized by volcano plots (Supplementary Figure 4). There were 550 (134 up-regulated) significantly differential metabolites between GF and WF, 504 (182 up-regulated) between GF and YF, 445 (258 up-regulated) between GF and PF, 542 (164 up-regulated) between PF and WF, 561 (194 up-regulated) between PF and YF, and 579 (151 up-regulated) WF and YF. The classification of the differential metabolites in the comparison between PF and YF showed that many flavonoids, organic acids, and lipids were up-regulated in PF (Supplementary Figure 5A). Classifying the differences between PF metabolites and WF metabolites (Supplementary Figure 5B) indicated that PF had more flavonoids, organic acids, nucleotides, and derivatives, as well as amino acids and derivatives compared to WF. Differential metabolites classification of the between PF and GF showed that flavonoids, organic acids, amino acids, and derivatives, as well as alkaloids were upregulated in PF (Supplementary Figure 5C).

### 3.4. KEGG pathway annotation for the differential metabolites

**FIGURE 5:**
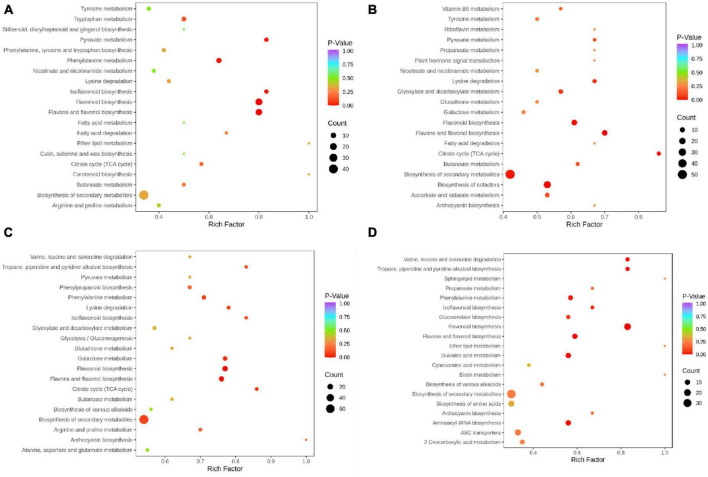
KEGG annotations and enrichment results of the differentially expressed metabolites in the pairwise comparison between: **(A)** PF and YF; **(B)** PF and WF; **(C)** PF and GF; and **(D)** YF and WF.

To detect the differential metabolites at gene expression levels, we performed KEGG pathway analysis. The KEGG pathway enrichment analysis revealed that flavonoids biosynthesis, flavone and flavonol biosynthesis, isoflavonoids biosynthesis, phenylalanine metabolism, and pyruvate metabolism were the main enrichment of the differential metabolites between PF and YF (Figure 5A). In addition to these metabolic pathways, the differential metabolites between the PF and WF were involved in the citrate cycle, as well as flavone and flavonol biosynthesis (Figure 5B). The differential metabolites between PF and GF were observed mostly in isoflavonoids biosynthesis, citrate cycle, lysine degradation, flavonoids biosynthesis, flavone, and flavonol biosynthesis (Figure 5C). Figure 5D indicated that the differential metabolites between YF and WF were enriched mainly in flavonoids biosynthesis, and valine, leucine and isoleucine degradation, and tropane, piperidine, and pyridine alkaloid biosynthesis. As can be seen in Supplementary Figure 6A, the differential metabolites between GF and YF occurred mainly in valine, leucine and isoleucine degradation, tropane, piperidine, and pyridine alkaloid, flavonoids biosynthesis, flavone, and flavonol biosynthesis. The differential metabolites between GF and WF were mainly involved in flavonoids biosynthesis, isoflavonoids biosynthesis, and phenylpropanoid biosynthesis (Supplementary Figure 6B).

### 3.5. Specific differential metabolites

**FIGURE 6:**
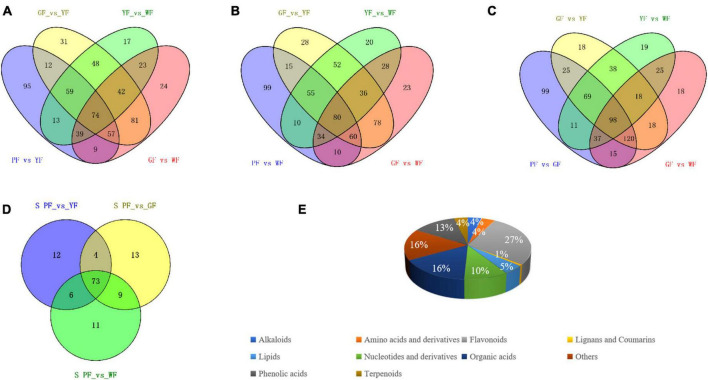
Identification and classification of key differently regulated metabolites in carnation flowers of different colors. **(A–C)** Venn diagram showing the number of the key metabolites that varied with the flower color change; and **(D)** the total number of 128 specific differential metabolites in PF vs. YF, PF vs. WF, and PF vs. GF. **(E)** Classification of the 128 specific differential metabolites.

To order to identify key metabolites that changed with flower color from purple to white, a Venn diagram was constructed among the differential metabolites between PF vs. YF, PF vs. WF, PF vs. GF, and GF vs. YF, YF vs. WF, and GF vs. WF, respectively (Figure 6). We compared the PF vs. YF, GF vs. YF, YF vs. WF, and GF vs. WF, and detected 95 specific differential metabolites in PF vs. YF (Figure 6A). The 99 metabolites in PF vs. WF were specific to PF vs. WF, GF vs. YF, YF vs. WF, and GF vs. WF (Figure 6B). The 99 specific’s metabolites in PF vs. GF metabolites were detected among PF vs. GF, GF vs. YF, YF vs. WF, and GF vs. WF (Figure 6C). The result showed that there were a total of 128 specific differential metabolites between PF vs. YF, PF vs. WF, and PF vs. GF (Figure 6D and Supplementary Table 3). The specific differential metabolites were categorized into 10 categories, among which flavonoids (27%), organic acids (16%), phenolic acids (13%), and nucleotides and derivatives (10%) were found to be the dominant metabolites (Figure 6E).

**FIGURE 7:**
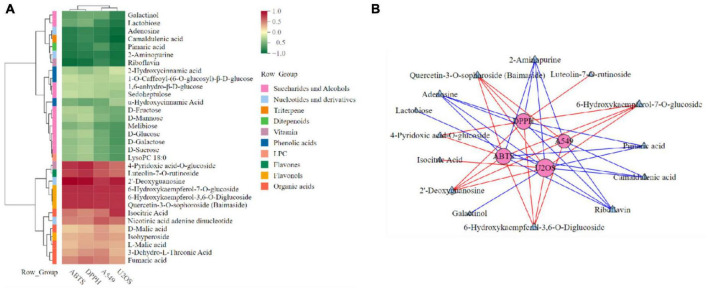
Spearman’s analysis and associated network diagram show the correlation between metabolites and four functional activities. **(A)** Spearman’s analysis of specific differential metabolites. **(B)** Associated network of specific differential metabolites.

**FIGURE 8:**
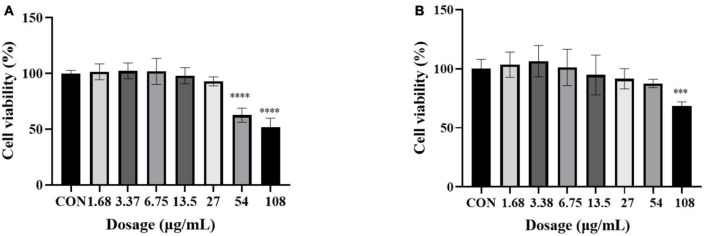
Cell viability of 2′-deoxyguanosine against A549 **(A)** and U2OS **(B)** cancer cell lines. Each assay was performed in triplicate. The drug group was compared with the control group, ^***^P < 0.001, ^****^P < 0.0001.

**FIGURE 9:**
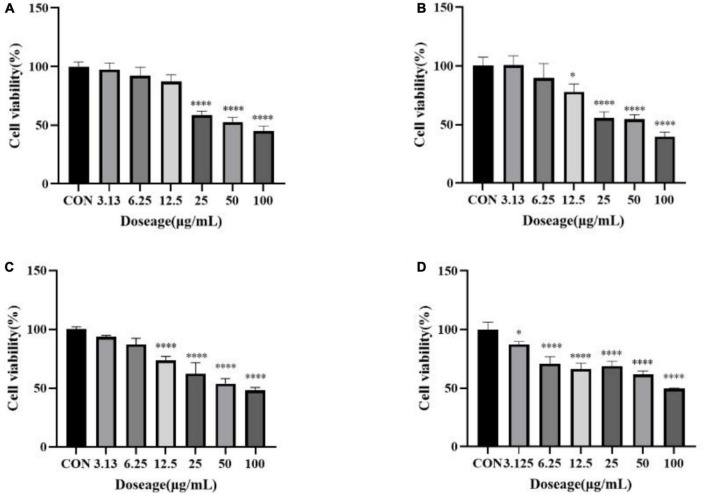
**(A)** Inhibition of a mixture of quercetin-3-*O*-sophoroside and 2′-deoxyguanosine on A549; **(B)** inhibition of a mixture of 6-hydroxykaempferol-3,6-*O*-diglucoside and 2′-deoxyguanosine on A549; **(C)** inhibition of a mixture of quercetin-3-*O*-sophoroside and 2′-deoxyguanosine on U2OS; **(D)** inhibition of a mixture of 6-hydroxykaempferol-3,6-*O*-diglucoside and 2′-deoxyguanosine on U2OS. The drug group was compared with the control group, *P < 0.05, ^****^P < 0.0001.

In order to further explore the correlation between metabolites and functional activity, a series of Spearman’s correlation analyses (*r* > 0.7 and <−0.7, *P* < 0.05) were performed and illustrated by heatmaps and network diagrams in Figure 7. The compounds with confidence levels 1 in 128 specific differential metabolites were selected that had a match score higher than 0.7 in secondary mass spectrometry and retention times of the metabolites and the database. A few of metabolites that correlated with antioxidant and anticancer abilities belonged to the upregulated metabolites (Figure 7A). Figure 7B further illustrated the complex relationships between these indicators and key metabolites. Correlation analyses revealed that the 6-hydroxykaempferol-3,6-*O*-diglucoside, 6-hydroxykaempferol-7-*O*-glucoside, quercetin-3-*O*-sophoroside, and 2′-deoxyguanosine were the major contributing factors for the four functional activities including antioxidant and anticancer, of which inhibitory activities of the A549 and U2OS cancer cell lines showed the higher coefficient correlations with the 2′-deoxyguanosine (*r* = −0.73 and −0.83, *P* < 0.01). 4-Pyridoxic acid-*O*-glucoside exhibited significant correlations with the antioxidant activities (*P* < 0.01), and luteolin-7-*O*-rutinoside exhibited significant correlations with the DPPH radical scavenging ability. Isocitric acid was involved in the anti-U2OS cancer cells. In addition, we investigated the effects of 2′-deoxyguanosine on cancer cell lines. The different concentration of 2′-deoxyguanosine were subjected for CCK-8 assay, as shown in Figure 8, indicating that 2′-deoxyguanosine has anticancer activity against the human A549 and U2OS cancer cell lines. Therefore, 2′-deoxyguanosine has potential as a useful source of anticancer drugs. In contrast, single quercetin-3-*O*-sophoroside and 6-hydroxykaempferol-3,6-*O*-diglucoside had no inhibitory effect on A549 and U2OS cells (Table 2). Because synergies can make the addition or mixing of two or more ingredients produce an effect greater than the effects of the ingredients applied separately. The inhibitory effects of quercetin-3-*O*-sophoroside mixed with 2′-deoxyguanosine and 6-hydroxykaempferol-3,6-*O*-diglucoside mixed with 2′-deoxyguanosine on A549 and U2OS cells were detected by CCK-8 method. The results showed that the synergistic drugs had better inhibitory effect on tumor cells than single compounds (Table 2 and Figure 9).

**TABLE 2:** IC_50_ values of different compounds in carnation.

No.	Sample	A549⋅IC_50_ (μ g/ml)	U2OS⋅IC_50_ (μ g/ml)
1	2′-Deoxyguanosine	≥108	≥150
2	Quercetin-3-*O*-sophoroside	–	–
3	6-Hydroxykaempferol-3,6-*O*-diglucoside	–	–
4	Quercetin-3-*O*-sophoroside mixed with 2′-deoxyguanosine (1:1)	61.46 ± 6.737	70.97 ± 17.19
5	6-Hydroxykaempferol-3,6-*O*-diglucoside mixed with 2′-deoxyguanosine (1:1)	53.82 ± 12.36	115.4 ± 5.82
6	Cisplatin (positive control)	15.86 ± 4.165	13.92 ± 1.542

The symbol “–” indicates no antitumor activity at experimental concentrations.

## 4. Discussion

There is a positive correlation between flower color and the antioxidant activity of flowers. The purple chrysanthemum tea was found to have greater antioxidant activity than yellow chrysanthemum tea in the DPPH radical scavenging assay (23). There was a significant and positive relationship between high levels of TPC (total phenolic content), TFC (total flavonoid content), TAC (total anthocyanin content), and antioxidant activity in the petals of *Malus* spp. during flower development (24). The purple petal extract of *P. delavayi* had the highest total phenolic content, total flavonoid content, and the strongest ABTS radical scavenging ability, the ferric reducing antioxidant potency compared to the red petal extract and yellow petal extract, which suggesting the anthocyanin biosynthesis, flavone and flavonol biosynthesis pathways are the key pathways responsible for both petal color and bioactive phytochemicals in *P. delavayi* flowers (25). The carnation flowers are purple to green in color. In the current study, we compared metabolome profiling of purple, green, yellow, and white carnation flowers and identified key metabolites that are responsible for antioxidant and anticancer abilities. We found that flower color of carnations is associated with antioxidant and anticancer activity. Purple carnation flowers had the highest antioxidant and anticancer properties.

Metabolomics analysis plays a key role in revealing the metabolic differences between the different colored flowers, and probiotic fermentation and in the elucidation of the relationship between bioactivities and phytochemicals (25, 26). There is little information on carnation metabolite diversity in different flowers colors. Zhang et al. (27) found the highest levels of pelargonidin 3-*O*-glucoside content in the petals of open flowers, and suggested that these anthocyanins were the major compounds in the red coloration of the petal margins. Dihydroflavones, flavonoids, and dihydroflavonols probably caused carnation petals to rang in coloration from white-green to yellow with development (27). Cyanidin 3,5-*O*-diglucoside, malvidin 3,5-diglucoside, and cyanidin 3-*O*-galactoside have been shown to be primarily responsible for the purple flower color of *Salvia miltiorrhiza* (28). In the case of *Phalaenopsis*, the combination of anthocyanin and/or carotenoid may determine petals color (29). A total of 932 metabolites were identified in the WF, YF, GF, and PF samples. Heatmap cluster and pearson correlation analysis grouped the WF, YF, GF, and PF samples. OPLS-DA revealed significant differences in metabolite levels between the four groups. A total of 445 metabolites were found to be significantly differentially expressed between GF and PF, 542 between WF and PF, and 561 between YF and PF. The metabolites identified in this study could be considered discriminatory biomarkers for future carnation flower studies. Purple carnation flowers had relatively high levels of flavonoids and organic acids. These results indicated that the biosynthesis pathways of flavonoid and organic acids may be more activated in purple carnation flower compared to light-colored flowers. Most of the specific differential metabolites identified, such as 6-hydroxykaempferol-3,6-*O*-diglucoside, 6-hydroxykaempferol-7-*O*-glucoside, quercetin-3-*O*-sophoroside and 2′-deoxyguanosine et al., had a high relative content in purple flowers and were found to have strong antioxidant and anticancer abilities. It can thus be used as a functional food with presumed pharmacological profits and can be deeply exploited for development of nutraceuticals-rich flower in carnation breeding.

The biosynthesis of the 2′-deoxyribosyl groups occurs solely by reduction of the 2′-hydroxyl group of di- or triphosphates of the ribonucleoside, catalyzed by ribonucleotide reductases (30, 31). Some organisms also possess deoxynucleoside kinases, which provide a salvage pathway for the use of preformed deoxynucleosides as a precursor of DNA. Since the cytotoxicity of a wide variety of deoxynucleoside analogs is dependent on the conversion of these compounds to the corresponding deoxynucleotide analog, substantial attention has been given to the substrate specificity properties and the regulation of various sources of deoxynucleoside kinases (32). Nucleoside analogs are widely used in anti-viral and anticancer therapies. Synthesis of natural analogs of deoxyribonucleosides is a successful strategy for finding inhibitors of viral and cellular DNA replication (33). The highly efficient phosphorylation of 2′-deoxyguanosine can be catalyzed by deoxyguanosine kinase (34). In current experiments, 2′-deoxyguanosine has been shown to have anticancer activity against the human A549 and U2OS cancer cell lines. To be best of our knowledge, this is the first report to show that 2′-deoxyguanosine could be a useful chemopreventive and/or chemotherapeutic agent for cancer.

Quercetin and its derivatives have diverse biological activities such as anticancer, anti-viral, and anti-oxidant properties (35). Kaempferol and its derivatives possess a wide range of therapeutic properties including antioxidant, anti-inflammatory, anticancer, antidiabetic, and anti-aging properties (36, 37). Quercetin and 6-hydroxykaempferol in ethanol extract of marigold (*T. erecta*) have been shown to have significant anticancer activity against both A549 and HEPG2 cells (38). Quercetin-3-*O*-sophoroside is an important member of the glucoside group of quercetin and is an established anti-inflammatory, antioxidant, anticancer, and antiviral edible compound (39). In this study, single quercetin-3-*O*-sophoroside and 6-hydroxykaempferol-3,6-*O*-diglucoside have no activities against both A549 and U2OS cells. However, flavonol glycosides screened from differential metabolites were used in combination with 2′-deoxyguanosine, which have anti-tumor activities. The combination of 2′-deoxyguanosine with 6-hydroxykaempferol-3,6-*O*-diglucoside or quercetin-3-*O*-sophoroside demonstrated synergism (Table 2).

Isocitric acid and its derivatives are used extensively in industries, pharmaceutical, and cosmetic production, and show activity superior to that of ascorbic acid in the model of oxidative-induced stress (40). Isocitric acid also reduces neurotoxicity through high concentrations of lead salt and molybdenum, in addition to the restoration of memory and acceleration of learning (41). It is possible that isocitric acid has anticancer activity against human U2OS cancer cell lines in the study and further studies are required.

## 5. Conclusion

In this study, we focused on the diversity and variability of metabolites in purple, yellow, white, and green carnation flowers, as well as antioxidant and anticancer activities to promote their overall use. We found that carnation flower metabolites diverged significantly with flower color change. The diversity of metabolites is largely determined by genotype. In total, we identified 128 key metabolites and pathways that could be associated with differences in antioxidant and anticancer activities. The significant differential metabolites between purple flowers and other groups mainly included flavonoids, organic acids, phenolic acids and, nucleotides and its derivatives. Flowers with purple carnations have better antioxidant and anticancer efficacy than flowers with other color. The biological characteristics of carnation flowers are thus primarily related to the content of flavonoids, organic acids, phenolic acids and, nucleotides and derivatives. This study demonstrated for the first time that 2′-deoxyguanosine has effective antiproliferative activity against human cancer cells A549 and U2OS. At the same time, the combination of 2′-deoxyguanosine with 6-hydroxykaempferol-3, 6-*O*-diglucoside or quercetin-3-*O*-sophoroside have also been found to increase the antitumor activity of 2′-deoxyguanosine. Its antitumor effect is close to that of purple carnation, which may be the material basis for the antitumor activity of purple carnation. However, due to the complex components contained in the extracts of purple carnation, its antioxidant and anti-tumor effects need to be further studied.

## Data availability statement

The original contributions presented in this study are included in the article/Supplementary material, further inquiries can be directed to the corresponding authors.

## Author contributions

XZ: formal analysis, methodology, investigation, and writing—original draft. MW and HL: investigation and validation. SY: writing—review and editing. WT: conceptualization, data curation, project administration, and methodology. All authors had read and agreed to the published version of the manuscript.

## Footnotes

1
www.r-project.org

2
http://www.kegg.jp/kegg/compound/
