# Supplementary figures and images for: Widely targeted metabolomics reveals the antioxidant and anticancer activities of different colors of Dianthus caryophyllus
Source: Front Nutr. 2023 May 19;10:1166375. doi: 10.3389/fnut.2023.1166375 (PMC10235515; doi:10.3389/fnut.2023.1166375)

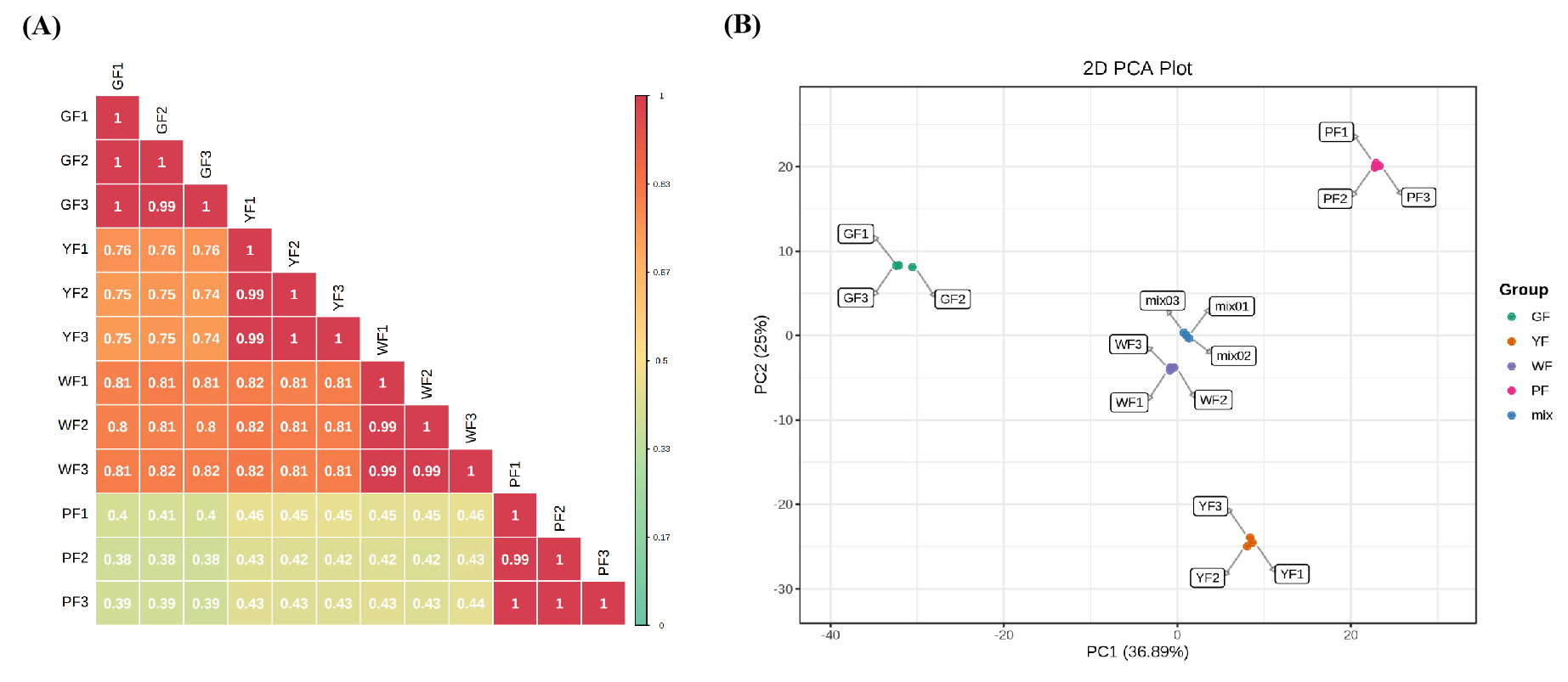

Supplement: Supplementary file 1 [file Image_1.TIF]

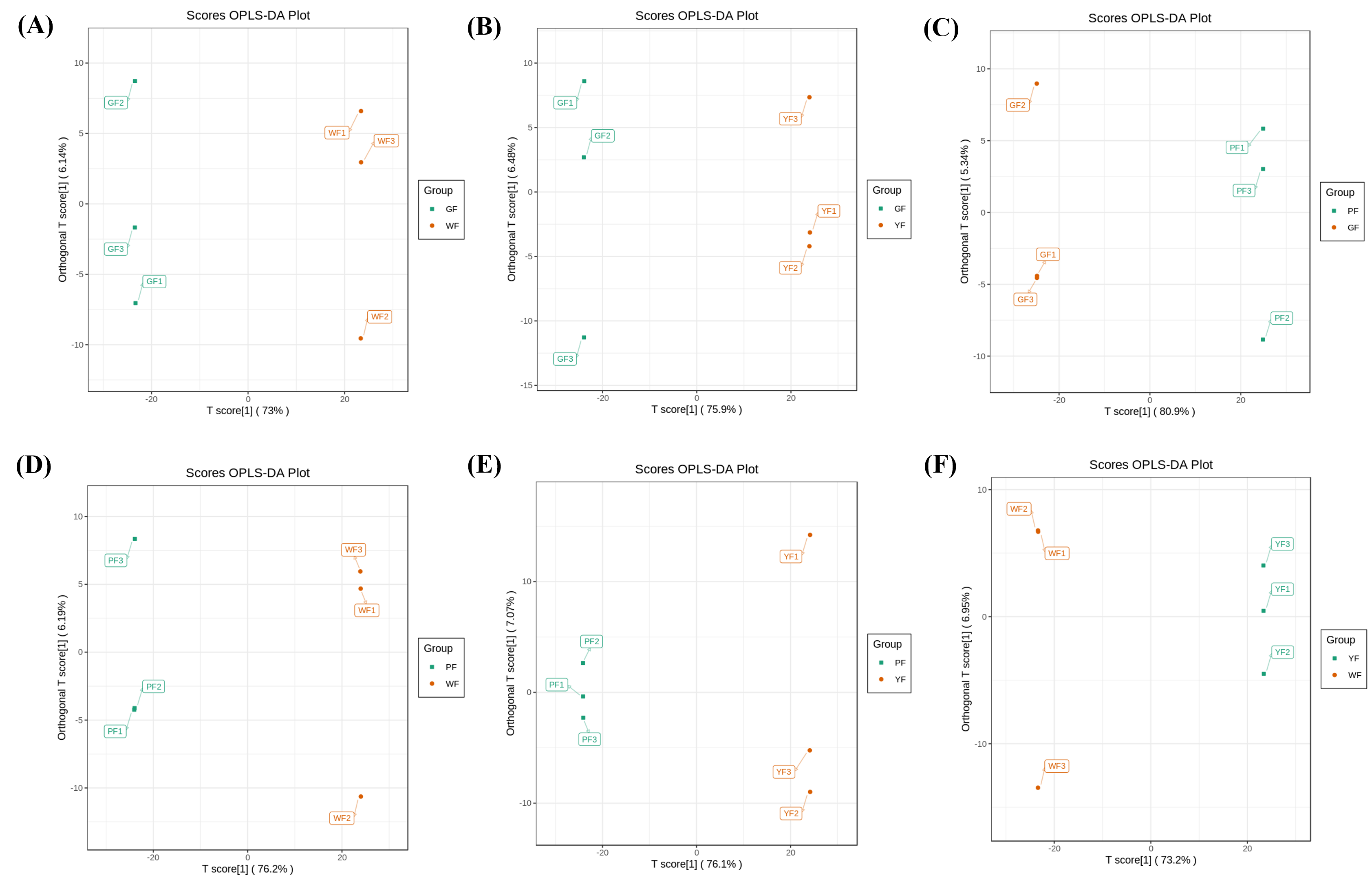

Supplement: Supplementary file 2 [file Image_2.TIF]

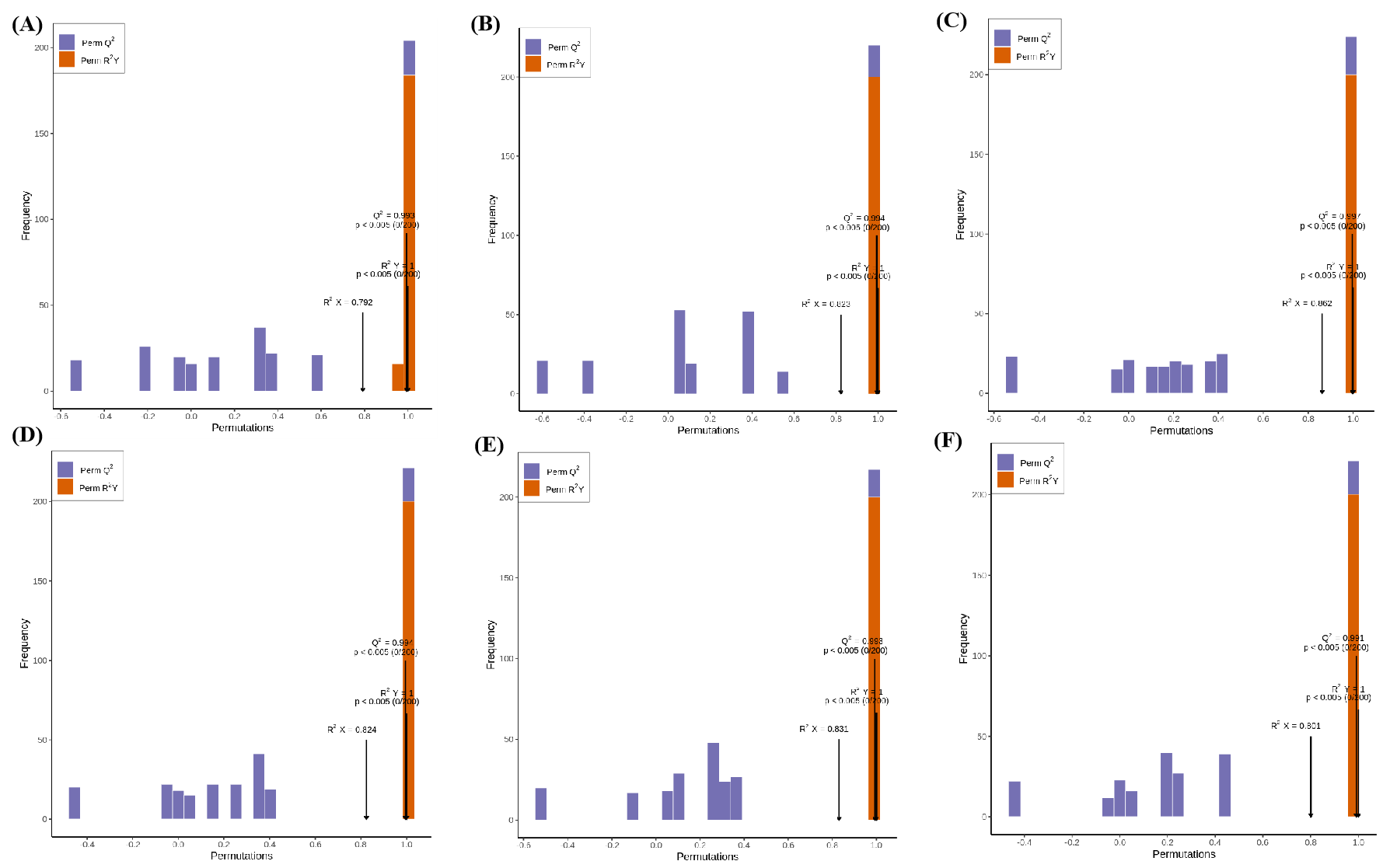

Supplement: Supplementary file 3 [file Image_3.TIF]

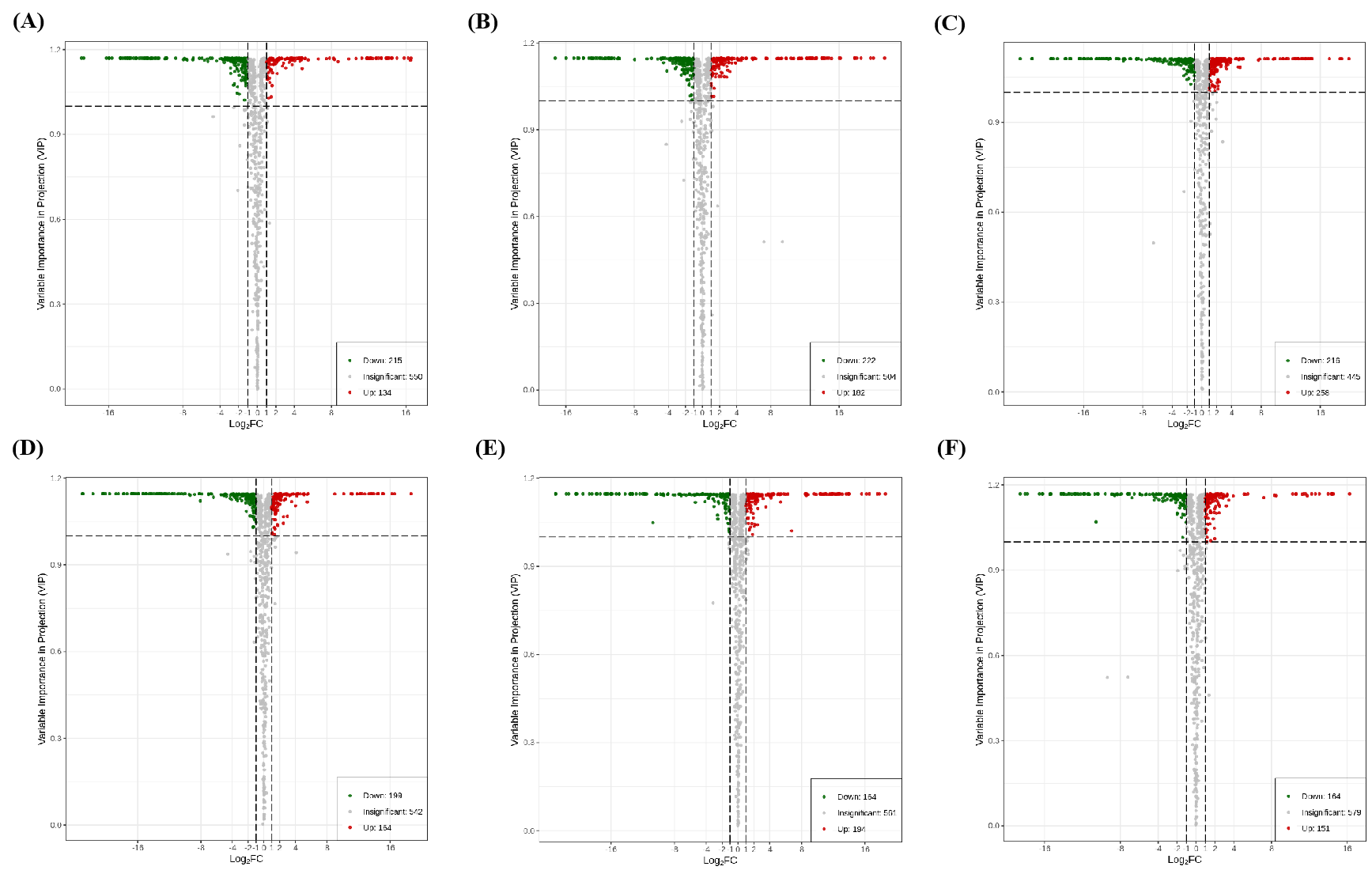

Supplement: Supplementary file 4 [file Image_4.TIF]

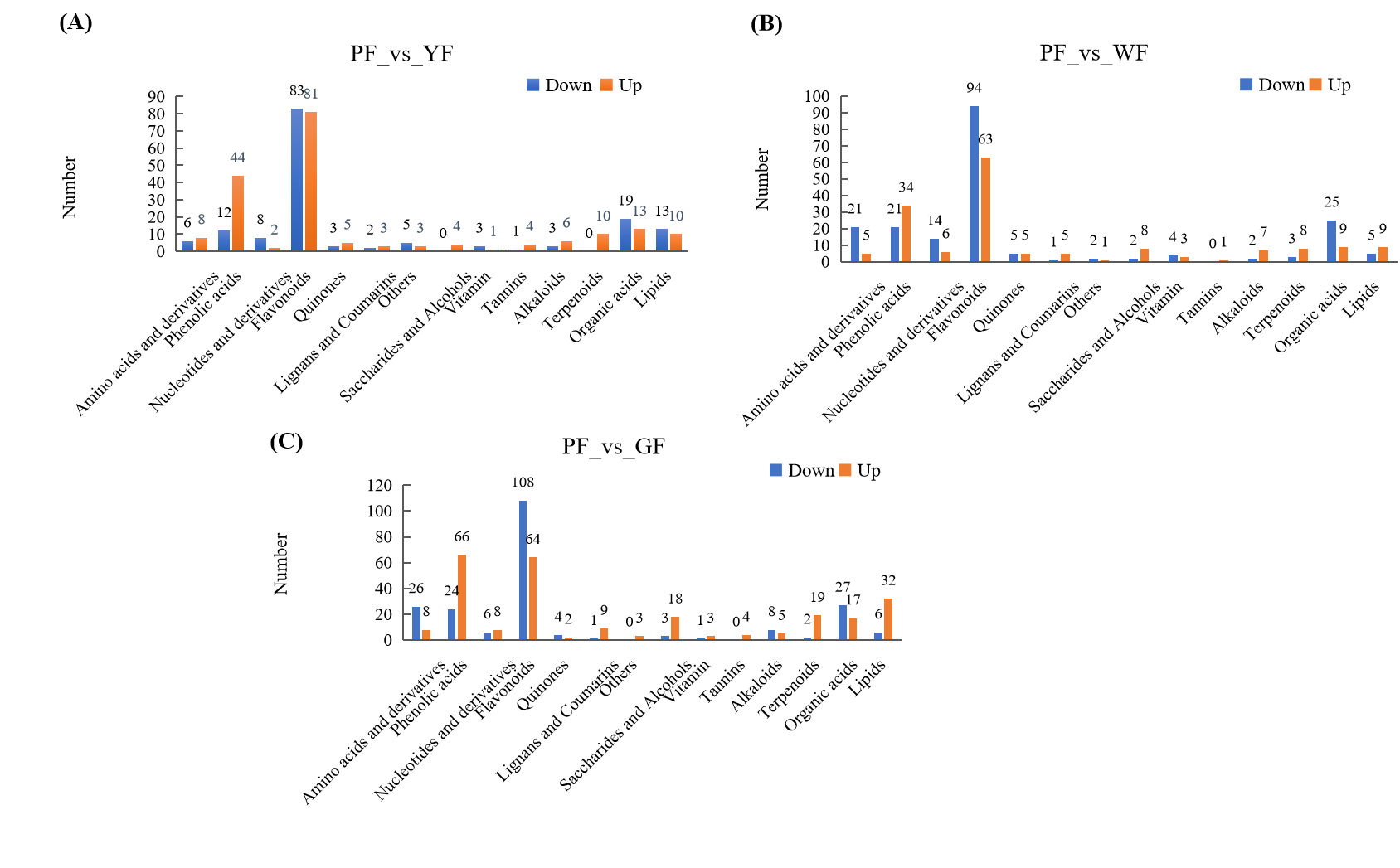

Supplement: Supplementary file 5 [file Image_5.TIF]

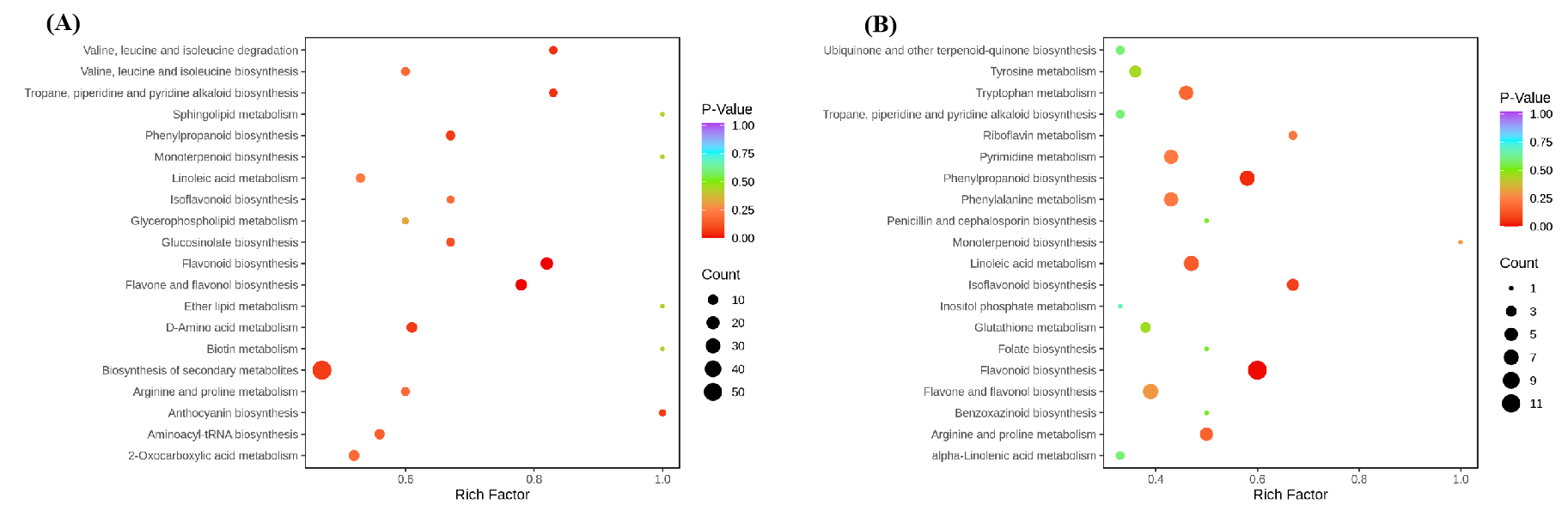

Supplement: Supplementary file 6 [file Image_6.TIF]
